# West Nile Virus Outbreak in Horses, Southern France, 2000: Results of a Serosurvey

**DOI:** 10.3201/eid0808.010486

**Published:** 2002-08

**Authors:** Benoit Durand, Véronique Chevalier, Régis Pouillot, Jacques Labie, Ingrid Marendat, Bernadette Murgue, Hervé Zeller, Stéphan Zientara

**Affiliations:** * Agence Française de Sécurité Sanitaire des Aliments, Maisons-Alfort, France; † Institut Pasteur, Paris, France

**Keywords:** West Nile Virus, equidae, France, seroepidemiologic studies, age factors, logistic regression

## Abstract

During late summer and autumn 2000, a West Nile fever outbreak in southern France resulted in 76 equine clinical cases; 21 horses died. We report the results of a large serosurvey of all equines within a 10-km radius of laboratory-confirmed cases. Blood samples were obtained from 5,107 equines, distributed in groups of 1 to 91 animals. West Nile virus immunoglobulin (Ig) G antibodies were found in 8.5% of animals (n=432). Forty-two percent of the IgG-positive animals were also IgM positive. Horses living in small groups were more affected than those in large groups. The results suggest that West Nile virus is not endemic in the affected area, the Camargue; rather, sporadic outbreaks are separated by long silent periods.

*West Nile virus* (WNV) is an arbovirus of the genus *Flavivirus*, family *Flaviridae*. Its natural transmission cycle involves birds and mosquitoes, mainly of the *Culex* genus. During late summer and autumn 2000, a WNV outbreak in southern France resulted in 76 clinical cases in equines; 21 horses died (1). The cases occurred near the Camargue region, a large wet area that corresponds to the delta of the Rhône River (Figure 1), near the Mediterranean coast. The area has a rich avifauna (2,3); >300 bird species, mostly water birds have been observed there. Among these species, some are migratory: Camargue is an important resting area for birds migrating between western Africa and northern Europe. Camargue is also a breeding area for some species and a wintering area for others. Mosquito density is high in this wet area (3,4). Among *Culex* species, *C. pipiens* and *C. modestus* are the most abundant.

**Figure 1:**
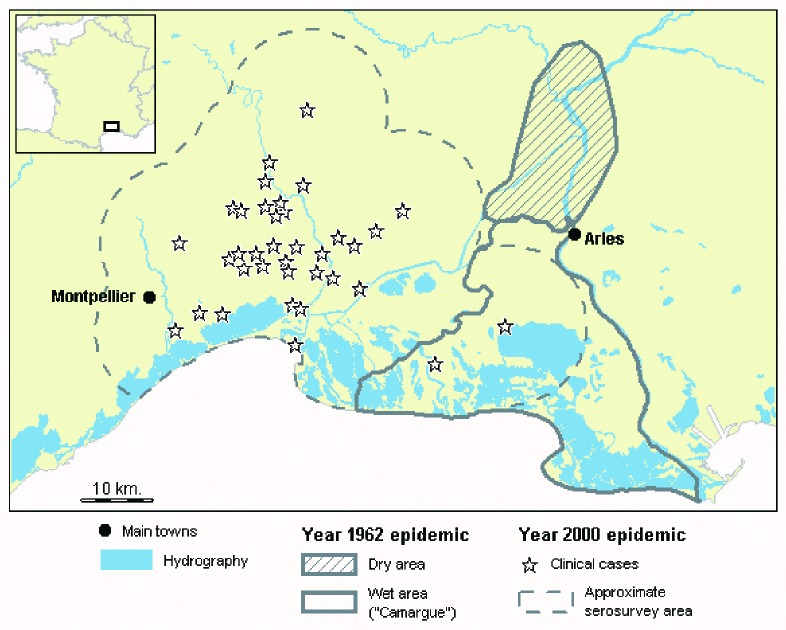
West Nile virus clinical infection in equines in southern France, 1962–2000.

In France, the first reported outbreak occurred in humans and equines during the summer of 1962 in the south of the country (5,6). Equine cases occurred both in Camargue (approximately 30 cases) and in a neighboring dry area (Figure 1; approximately 50 cases). From 1963 through 1964, a serosurvey was conducted in both areas: 6 of 37 horses were found positive for WNV (6).

The 2000 outbreak occurred west of Camargue (Figure 1), where the landscape features two very different biotopes. The coast is mainly wet areas with rice fields, numerous ponds, and marshes. North of these wet areas are dry areas with vineyards, farming areas, and typical Mediterranean vegetation. Most of the cases occurred in the dry areas (Figure 1).

On September 6, 2000, positive serologic results (immunoglobulin [Ig] G and IgM) were first found in two horse samples. Two days later, WNV infection was confirmed by detection of viral RNA in a brain biopsy (1). Clinical cases were observed until November 2. No abnormal deaths were observed in birds, and a serosurvey was conducted in November and December 2000 with captive ducks and wild birds (sparrows, gulls, and magpies). Positive results were found in one gull, eight ducks, and four magpies (7). Mosquitoes were also collected in the outbreak area, but none of the pools was found positive. No human cases were reported; however, WNV neutralizing antibodies were detected in three gamekeepers working in the area, one of whom also had IgM antibodies (1).

Experimental studies and sequential samples collected from naturally infected horses have shown that IgM antibodies become detectable 8–10 days post-infection and persist <2–3 months (8,9). WNV neutralizing antibodies can persist >2 years after infection (9). No published data could be found about the evolution of the WNV IgG response in horses; however, IgG neutralizing antibodies may persist several years after infection.

After the first horse case was confirmed, a serosurvey was ordered by the animal health authorities on all equines located within a 10-km radius of laboratory-confirmed clinical cases (1). Preventive measures included prohibiting movements of horses inside this perimeter. We report the results of this serosurvey, the first large-scale serosurvey conducted in equines worldwide.

## Material and Methods

Blood samples were taken from all the equines within 10 km of the laboratory-confirmed cases (Figure 1). The use of a 10-km radius area for control measures is common in animal diseases control plans (e.g., against foot and mouth disease or classical swine fever). The sera were processed and tested for WNV IgG and IgM antibodies as described (1). Animals were first tested for WNV IgG antibodies, and because of logistic constraints, only positive sera were then tested for IgM antibodies. A positive animal was defined as an IgG-positive animal. A positive group was defined as a group in which at least one animal was IgG positive.

For each animal, a form was completed by a veterinarian. Along with other data (date, names and addresses of the veterinarian and the animal caretaker), this form noted the species and breed of the animal, its age and sex, location, and the size of the group in which the animal was included on the day the sample was taken (i.e., the number of equines at the same place).

The individual serologic results and the data on the forms were collected in a Microsoft Access database (Microsoft Corp., Redmond, WA). The horses were grouped by sex (geldings, mares, and stallions) and age (<1 year, 1–2 years, 3–5 years, 6–10 years, 11–15 years, 16–20 years, 21–25 years, and >25 years). Breeds were grouped into four classes according to their typical management conditions: the pure breeds (expensive animals kept in stalls at night and pastures by day), the Camargue breed (an ancient breed of rugged saddle horses or half-wild animals originating in the survey area, always kept outside), pony breeds (often living in riding stables and used every day for short rides) and other breeds (donkeys and other equines, typically kept outside in small groups on pastures). Four frequency categories (all having the same number of animals) were defined for group sizes: 1–2 animals, 3–6 animals, 7–25 animals, and >25 animals.

Maps were produced with MapInfo software (MapInfo Corp., Troy, NY). The geographic data describing animal locations was the name of the “commune,” the smallest administrative French subdivision, which corresponds to an English parish.

Statistical analyses were done with SAS software (SAS Institute Inc., Cary, NC). A bivariate analysis was first performed, crossing each variable (age, sex, breed, and group size). The chi square test was calculated for the four variables. Prevalence ratios were computed for breed and group size, using the category with the lowest prevalence as a reference. For animals groups, chi square tests and prevalence ratios were computed both at the animal level (crossing the group size class with the animal-level prevalence) and the group level (crossing the group size class with the group-level prevalence). Finally, a logistic regression was conducted. Animals for which the age, sex, breed, or group size had not been indicated were excluded from the data set. The dependent variable of the model was the serologic status of animals, and the independent variables were their age class, sex, breed, and group size. (The location of animals could not be included in the model because many regression classes would have been empty.) For each variable, the reference class was the category with the lowest prevalence.

## Results

### Descriptive Analyses

Fifty veterinarians took blood samples from 5,107 equines (4,776 horses and 91 donkeys) distributed in 1,429 groups. The 14-week serosurvey began on September 16 (week 38) and ended December 15. The survey area was approximately 2,500 km^2^ and covered (partially or totally) 99 communes.

The age of 4,749 animals ranged from birth to 40 years. Half the animals were geldings (48%, n=2,345), 32% were mares (n=1,533), and 20% were stallions (n=951). Of 53 breeds noted for 4,867 animals, the predominant breed was the Camargue (36%; n=1,743). Group sizes ranged from 1 to 91 animals, but most of the groups (70%; n=1,006) were small (1–2 animals). The mean group size was 3.6 animals.

Of the 5,107 animals, 432 were IgG positive (8.5%; 95% confidence interval [CI] 7.7 to 9.2). Almost half (n=182) were also IgM positive (42.1%; 95% CI 40.8 to 43.5). The group-level seroprevalence of the positive groups was higher than the animal-level seroprevalence: 19.2% (n=274) (95% CI 17.1 to 21.2).

### Time Distribution

**Figure 2:**
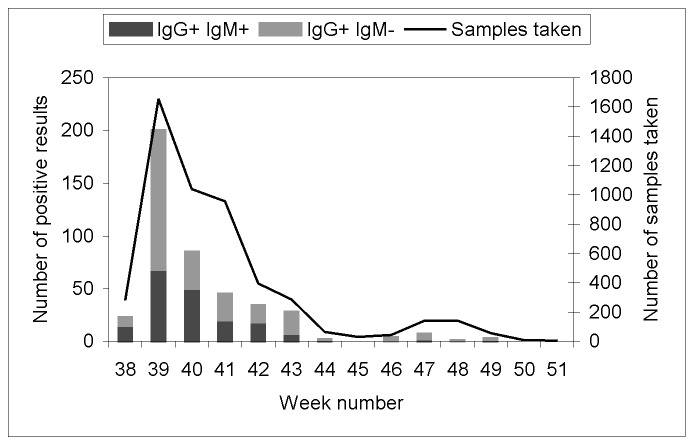
West Nile virus serosurvey, Camargue, France, 2000: number of samples and seropositive animals, by week.

More than 50% of the samples were taken during the first 3 weeks; 90% of the samples had been taken at the end of week 6 (Figure 2). IgG-positive animals were identified throughout the 14-week study period: the last positive animals were found during week 12. Animals that were both IgG- and IgM-positive were also found until week 12, with a slight decrease over time (15 of 23 during week 1 and 1 of 3 during week 12).

### Geographic Distribution

**Figure 3:**
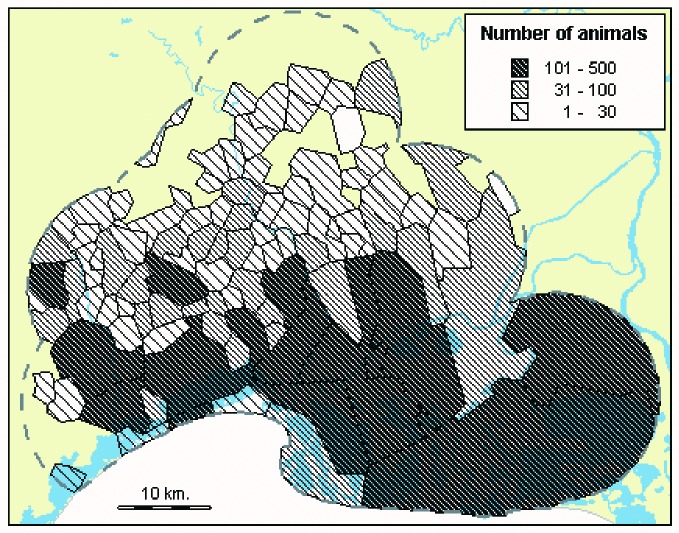
West Nile virus serosurvey, Camargue, France, 2000: geographic distribution of the animals sampled, by commune (n=5,905).

The map of the number of serum samples per commune shows that the geographic distribution of horses is not homogeneous (Figure 3): fewer samples were taken from horses in the north of the survey area, where land is more devoted to vineyards and agriculture, than in the south, in or near wet areas. The overall animal density is approximately 2 animals per km^2^. However, the density in some communes is >16 animals per km^2^.

**Figure 4:**
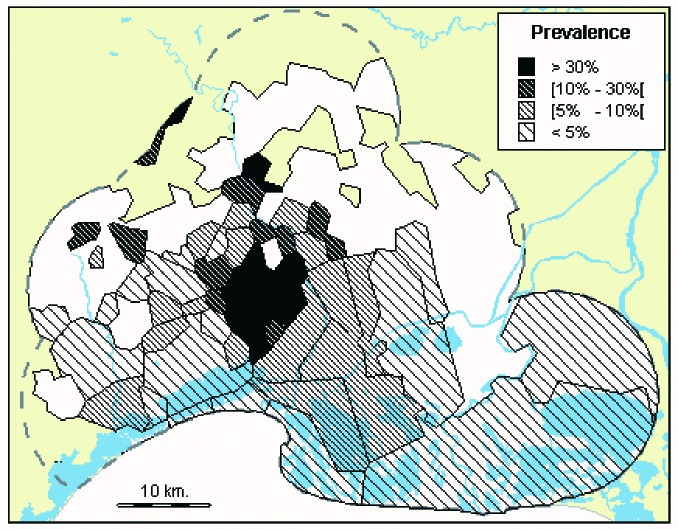
Immunoglobulin G anti-West Nile virus prevalence in 5,095 equines, by commune, Camargue, France, 2000 (n=5,095).

The prevalence by commune (Figure 4) is higher in the center of the area, as is also the case for the geographic distribution of clinical cases (Figure 1). The main part of this “hot spot” is not in a wet area, but in a rather dry area. In this focal point, the prevalence exceeds 30% in eight communes and reaches 58% in one (28 positive of 48 animals). A similar pattern is found for the group-level seroprevalence, with higher rates, exceeding 50% in nine communes and reaching 88% in one, with 21 positive groups of 24.

### Bivariate Analysis

No significant difference was found in the serologic status of animals according to their distribution by age (chi square 6.63; p=0.47; Table 1) and by sex (chi square 2.65; p=0.27). A significant difference was found in the serologic status of animals by breed (chi square=11.12; p=0.01; Table 2), with a prevalence ratio of 1.5 (95% CI 1.2 to 1.9) for the Camargue breed. A significant difference was also found in the serologic status of animals by group size (chi square 54.11; p<0.0001; Table 3), and decreasing prevalence ratio was observed as group size increased. At the group level, a significant difference was also found in the serologic status of groups according to their distribution by size (chi square 55.68; p<0.0001; Table 4); however, increasing prevalence ratios were observed with increasing group size.

**Table 1:** Immunoglobulin G anti-West Nile virus prevalence in equines by age group, Camargue, France, 2000^a^

Age (yrs)	Total	Positive (%)
<1	33	2 (6.1)
1–2	368	44 (12.0)
3–5	938	88 (9.4)
6–10	1,559	124 (8.0)
11–15	975	87 (8.9)
16–20	555	50 (9.0)
21–25	229	21 (9.2)
>25	92	9 (9.8)
Total	4,749	425 (8.9)

^a^Chi square = 6.63 (7 degrees of freedom); p=0.47.

**Table 2:** Immunoglobulin G anti-West Nile virus prevalence in equines by breed, Camargue, France, 2000^a^

Breed class	Total	Positive (%)	Prevalence Ratio	95% CI
Pure breeds	1,252	108 (8.6)	1.2	0.9 to 1.7
Camargue	1,743	183 (10.5)	1.5	1.2 to1.9
Ponies	497	38 (7.6)	1.0	0.7 to1.5
Other	1,375	100 (7.3)	Ref.	
Total	4,867	429 (8.8)		

^a^Chi square=11.12 (3 degrees of freedom), p=0.01; 95% CI = 95% confidence intervals.

**Table 3:** Immunoglobulin G anti-West Nile virus individual-level prevalence in equines by group size, Camargue, France, 2000^a^

Group size	Total	Positive (%)	Prevalence Ratio	95% CI
1–2 animals	1,353	165 (12.2)	2.2	1.6 to 3.0
3–6 animals	1,118	114 (10.2)	1.8	1.3 to 2.5
7–25 animals	1,355	87 (6.4)	1.1	0.8 to 1.5
>25 animals	1,281	55 (5.1)	Ref.	
Total	5,107	432 (9.7)		

^a^ Chi square = 54.11 (3 degrees of freedom), p<0.0001; 95% CI = 95% confidence intervals.

**Table 4:** Immunoglobulin G anti-West Nile virus group-level prevalence in equines by group size, Camargue, France, 2000^a^

Size class	Total	Positive (%)	Prevalence Ratio	95% CI
1–2	1,006	149 (14.8%)	Ref.	
3–6	294	73 (24.8%)	1.9	1.4-2.6
7–25	98	40 (40.8%)	4.0	2.6-6.1
>25	31	12 (38.7%)	3.6	1.7-7.6
Total	1,429	274 (19.2%)		

^a^ Chi square = 55.68 (3 degrees of freedom), p<0.0001.

### Logistic Regression Results

The data set used for the logistic regression (animals for which the age, sex, breed, and group size were known) contained 4,597 records. The reference classes were as follows: <1 year, mares, other breeds, and >25 animals. A slight effect was found for the Camargue breed (odds ratio [OR] 1.40; 95% CI 1.08 to 1.82). Conversely, the main effect was attributed to the group size variable, with decreasing ORs with increasing group size: 2.18 for groups of 1–2 animals (95% CI 1.60 to 2.96) and 1.81 for groups of 3–6 animals (Table 5).

**Table 5:** Immunoglobulin G anti-West Nile virus prevalence in equines, by age, sex, breed, and group size, Camargue, France, 2000

Variable	OR estimate^a^	95% CI
Age (years)		
<1	Ref	
1–2	1.83	0.42 to7.99
3–5	1.39	0.32 to 5.98
6–10	1.13	0.26 to 4.86
11–15	1.25	0.29 to 5.41
16–20	1.24	0.28 to 5.43
21–25	1.20	0.26 to 5.48
>25	1.24	0.25 to 6.15
Sex		
Mare	Ref.	
Gelding	1.13	0.89 to 1.45
Stallion	1.18	0.88 to 1.58
Breed		
Pure breeds	1.30	0.97 to 1.74
Camargue	1.40	1.08 to 1.82
Ponies	1.15	0.77 to 1.72
Other	Ref.	
Group size		
1–2	2.18	1.60 to 2.96
3–6	1.81	1.31 to 2.52
7–25	1.09	0.77 to 1.53
>25	Ref.	

^a^OR, odds ratio; 95% CI, 95% confidence intervals.

Belonging to a small group and, to a lesser extent, being of the Camargue breed appeared to be risk factors for seropositivity. Comparison of the eight most affected communes with the rest of the survey area showed no significant differences by age, sex, breed, and group size (data not shown). Therefore, these risk factors are not explained by an overrepresentation of Camargue horses and of small groups near the epidemic hot spot.

## Discussion

### Serosurvey Results

The geographic variations of the seroprevalence show that the epidemic hot spot was not located in a wet area, but several km to the north, in a rather dry area, even though the horse density was roughly the same in both areas. Moreover, antibodies were found in 8% of the captive mallards at a large pond in the south of the perimeter, and in four magpies of 18 captured a few km to the north, near the horse epidemic hot spot (7). The existence and the specific location of the epidemic hot spot could therefore be explained by primary circulation of the virus in water birds in wet areas, followed by an amplification of this circulation by synanthropic bird species, involving spread from wet areas to dry areas, where these birds usually live.

Data analysis showed no age effect. Several serosurveys in human populations have shown that, when WNV circulation is endemic in a given area, the seroprevalence tends to increase with age (10–13). A similar result was found in Egyptian equines in 1963 (14). However, no such increase was found in 1999 in dogs in New York City, where the virus was thought to have been absent before that year (15). Assuming that anti-WNV IgG usually persists several years after infection and that most of the animals had lived in the survey area for a long time (and that all age classes are roughly equally exposed to infecting bites), these results suggest the absence of an endemic circulation of the WNV in the area. However, the existence of an endemic transmission cycle, geographically restricted to marshes (where half-wild horses live that could not be sampled) cannot be excluded.

A breed effect was observed in that the prevalence was higher in Camargue horses. This result reflects the usual living conditions of these rugged horses, always living outside and therefore more likely to be exposed to infectious bites.

Group size had two opposite effects on seroprevalence, depending on the unit used for calculation. At the group level, the increase in seroprevalence with increasing group size is a classical finding in veterinary epidemiology: assuming all animals in a given group are exposed to the same low-level probability of infection, the more animals in the group, the higher the probability that one (or more) of them become infected. At the individual level, the decrease of the seroprevalence rate with increasing group size may be the result of two factors. First, the sizes of the groups reflect different uses and management conditions of the horses: animals kept in large groups may benefit from better management practices (e.g., stabling at night, which could reduce their exposure to infecting bites). However, this result could also reflect a low spatial density of infectious vectors: assuming a vector does not bite all animals of a given group but only a few of them, large groups would have a protective effect. The high density of horses in the area could also help explain the absence of reported human cases as a result of a possible zooprophylactic effect of domestic animals, as pointed out by Komar et al. (15) (domestic animals may divert infectious bites from human hosts). Because of lack of data about the primary use and the management conditions of the horses, we could not evaluate the respective importance of these two factors on the protective effect of large groups.

### Limits and Biases

The survey was intended to be comprehensive for all equines located <10 km from laboratory-confirmed clinical cases. Movements of horses in and out of the area probably occurred at the beginning of the outbreak, before the restrictive measures taken by the animal health authorities were in place. Some of the Camargue horses are half-wild and live year-round in marshes; for practical reasons, these half-wild animals were not sampled. However, even if the survey was not strictly exhaustive, the 5,107-equine sample is certainly highly representative of the domestic equines in the area.

Having tested only the IgG-positive sera for IgM antibodies is an obvious bias: recently infected animals (with IgM antibodies but without IgG antibodies) that did not show clinical signs would have been missed. The seroprevalence figures obtained may be underestimated for domestic equines. Belonging to the Camargue breed was identified as a seropositivity risk factor and half-wild animals that were not sampled belong to this breed; therefore, the seroprevalence rate is probably also underestimated for the whole equine population of the area (domestic and half-wild equines).

The geographic distribution of the positive results shows that, in the east part of the survey area (and to a lesser extent the west), some positive results were still found near the area boundary: more positive results may have been included if the perimeter had been larger.

The serosurvey was carried out over a 14-week period; most of the samples were taken during the first 6 weeks. Because recent WNV circulation was detected (through IgM-positive results) until week 12, some animals tested at the beginning of the study were probably infected later, and the prevalence might have been higher if the study had been conducted during winter.

### Comparison with Other Studies

Few serosurveys have been conducted in equines. Survey results can be usefully compared between each other in disease-endemic areas (14); however, comparison is difficult if the survey is in epidemic areas. In such areas, the seroprevalence rate depends on the definition of the survey perimeter (which varies between studies). For example, in our study, if a radius of 15 km around cases had been used to define the survey perimeter instead of 10 km, the seroprevalence rate probably would have been lower.

In an endemic area, Egypt in 1959 (14), high seroprevalence rates were found, with figures ranging from 36% to 81%. Conversely, in a newly infected area, New York City in 1999 (15), a much lower rate was found: 2.7% of 71 horses (95% CI 0.3 to 9.5). The seroprevalence we observed is closer to the latter rate. However, in the Egyptian survey, the 15% seroprevalence rate in young horses (<2 yrs) is closer to our result: this figure better reflects the yearly infection rate and thus the infection pressure.

The seroprevalence rate can also be compared with the results of two earlier serosurveys conducted in the studied area. After the 1962 epidemic (Figure 1), in 37 sera collected in 1963–1964 from horses that did not have clinical signs the year before, 6 were positive (16.2%; 95% CI 4.3 to 28.8) (6). From 1975 to 1979, a low frequency of antibody response against WNV was observed in 99 horse samples (2%) (16). Therefore, in the studied region, WNV epidemics may occur sporadically and be followed by long silent periods.

The results of this study, the first large-scale WNV serosurvey conducted in equines, show that seroprevalence rate does not increase with age. Assuming that, as neutralizing antibodies, IgG antibodies persist several years after infection, this result (and the WNV history in the studied area since 1962) suggest that the Camargue is an epidemic area, with outbreaks that occur periodically and are separated by long silent periods. The seroprevalence level, especially for animal groups, indicates that virus circulation was intense during the 2000 epidemic. This intense virus circulation and the location of the epidemic focus in a dry area could be explained by the amplification by synanthropic bird species in dry areas, from a primary circulation in wet areas in water birds. The survey results do not allow us to assert whether this primary circulation is permanent (the virus being periodically reintroduced by migratory birds). However, the survey results suggest that, if the primary cycle is permanent, it is restricted to small marshy areas. Two further studies, a serologic follow-up of captive ducks and a serosurvey focused on horses living in the marshy areas, will be conducted in 2001–2002; these studies should allow us to refine the epidemiologic status of this primary cycle.
